# Effect of Implant Surface Decontamination Procedures on Surface Morphology—In Vitro Study

**DOI:** 10.3390/jfb17040166

**Published:** 2026-04-01

**Authors:** Furkan Özay, Selim Ersanlı

**Affiliations:** 1 Oral Implantology Program, Institute of Graduate Studies in Health Sciences, Istanbul University, 34098 Istanbul, Turkey; 2 Department of Oral Implantology, Faculty of Dentistry, Istanbul University, Fatih, 34452 Istanbul, Turkey

**Keywords:** electrolytic cleaning, electrolytic decontamination, dental implants, periimplantitis, GalvoSurge

## Abstract

Numerous chemical and physical surface decontamination methods are used in clinical practice for implant surface decontamination, which constitutes the most critical step in the management of peri-implantitis. The aim of this study was to compare, in vitro, the efficacy of the electrolytic cleaning device GalvoSurge (GalvoSurge, GalvoSurge Dental AG, Widnau, Switzerland) with that of an air-abrasive AIRFLOW unit (AIRFLOW, Master PiezonVR, EMS Electro Medical Systems, Herrliberg, Switzerland). Thirty-two SLA-surfaced dental implants were allocated to two groups (n = 16) and contaminated with permanent ink, after which they were placed into jaw models representing two different defect configurations. After treatment, implants were photographed and, using ImageJ, the residual stain area/percentage within a 4 mm region apical to the implant neck was calculated. Surface topography was further evaluated by SEM and EDS. In the two-way analysis of variance, the effect of the decontamination method was statistically significant. The GalvoSurge group exhibited a lower residual stain percentage than AIRFLOW (overall 28.47 ± 10.13 vs. 37.14 ± 9.60; *p* = 0.019). This difference was independent of defect type (*p* > 0.05). These findings indicate that electrochemical cleaning via galvanic current may be more effective, under in vitro conditions, for stain removal and surface decontamination; however, they also demonstrate that residual contamination could not be completely eliminated irrespective of the method.

## 1. Introduction

Dental implants are contemporary and highly predictable treatment modalities that help restore esthetic, mechanical, and phonetic functions that may be lost over time in the oral cavity. In implant therapy, successful early treatment is indicated by achieving primary and subsequently secondary osseointegration following implant placement in the jawbone. Long-term success, in turn, is reflected by adequate quantity and quality of peri-implant bone capable of supporting the forces transmitted to the implant via the prosthesis [1].

The presence of sufficient bone at the intended implant site within the jawbone is crucial for both short- and long-term success of implant therapy. For osseointegration to occur, implants must be surrounded by bone on all sides. If there is a region around the implant within the alveolar bone that lacks bony contact, this may partially or completely prevent early osseointegration. Such a condition can also pose a risk for long-term implant success [2].

After completion of osseointegration, a frequently encountered early or late biological complication is biofilm-associated peri-implant disease. These conditions are referred to as peri-implant mucositis and peri-implantitis [3].

Peri-implantitis can be defined as a pathological condition that develops around an osseointegrated implant in association with biofilm, characterized by bleeding on probing (BoP) and/or suppuration, followed by progressive bone loss [4].

In peri-implantitis therapy, mechanical biofilm removal can be performed using hand instruments such as steel, titanium, carbon-fiber, or plastic curettes, and/or ultrasonic devices with various tips, allowing removal of supra- and submucosal calculus and biofilm.

Mechanical cleaning of supra- and submucosal biofilm accumulations can be achieved by air-water spraying with an air-abrasive powder. Flexible plastic tips may be used to access submucosal biofilm; however, it is important to avoid surgical emphysema when attempting to reach deep peri-implant pockets. Meta-analyses have shown that non-surgical biofilm removal with powder-based air-water spray devices yields better outcomes in terms of bleeding on probing compared with mechanical debridement alone, adjunctive antiseptic therapy, or Er:YAG laser therapy [5].

Air-abrasive devices, one of the most commonly used implant surface decontamination methods, have been reported to be promising in clinical use based on plaque index and bleeding on probing measurements [6]. Despite their benefits, the literature also reports that these procedures may cause minor surface alterations and changes in the titanium coating [7].

In recent years, implant decontamination using devices operating with galvanic current has also been demonstrated in the literature [8]. Furthermore, these devices have been observed to induce favorable surface changes by increasing hydrophilicity, thereby potentially facilitating integration [9,10].

Before these methods can be implemented in routine clinical practice, they must first be evaluated in vitro. To compare clinical effectiveness, the AIRFLOW device (AIRFLOW, Master PiezonVR, EMS Electro Medical Systems, Herrliberg, Switzerland), which has been extensively studied in the literature, and the GalvoSurge device (GalvoSurge; GalvoSurge Dental AG, Widnau, Switzerland), which utilizes a contemporary technology based on electrical current, were selected for investigation. In studies on peri-implantitis therapy, comparisons between air-abrasive devices and electrolytic surface-cleaning systems remain limited [8,10].

The aim of this study was to compare, in vitro, electrical decontamination with air-abrasive procedures based on powder spraying. In addition, the study sought to contribute to peri-implantitis therapy by quantitatively measuring the cleaning capacity of these procedures and the associated morphological alterations on implant surfaces.

## 2. Materials and Methods

### 2.1. Ethics Committee Approval

As stated in the letter dated 8 March 2024 (no. 2459626) issued by the Department of Oral Implantology, Faculty of Dentistry, Istanbul University, our study (file no. 2024/22) was reviewed at the meeting of the Istanbul University Faculty of Dentistry Clinical Research Ethics Committee on 19 March 2024 (meeting no. 165) and was found ethically appropriate.

### 2.2. Determination of Sample Size

For the in vitro analysis, a power analysis was performed using G*Power (G*Power software, version 3.1.9.7, Düsseldorf, Germany) to determine the sample size. Study power is expressed as 1 − β (β = probability of a type II error).

In the literature review, similar studies were taken as examples [8,11,12,13,14].

Unlike those found in the literature, our study was designed to be conducted with two treatment groups. Based on values related to the investigated methods, the total required sample size calculated using the G*Power program was n = 30 with an effect size of 0.95, 80% power, and a 0.50 margin of error. Because the calculation was based on comparing two groups, the sample size was determined using an independent samples t-test. The study was designed with equal sample sizes per group; thus, the minimum sample size was set as n = 15 per group and at least n = 30 in total. Accordingly, it was decided to include 16 implants in each group in the present study. In addition, one implant was evaluated as a test implant without contamination and without applying any treatment method. This test implant was examined only under an electron microscope, and the results were photographed.

### 2.3. Preparation of In Vitro Models

**Figure 1:**
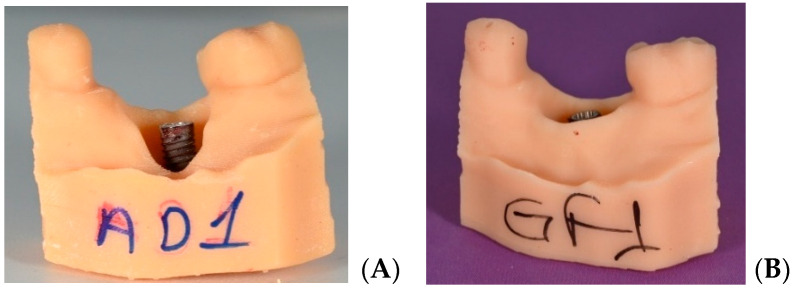
(**A**) Dehiscence type defect. (**B**) Fossa type defect.

In vitro hemimandible models were designed using EXOCAD DentalCAD v3.1 Rijeka software (exocad GmbH, Darmstadt, Germany). The jaw models were generated by selecting from ready-made templates. In this context, a left mandible-like model was created. In the design model, teeth 35 and 37 were designed as present, and an implant was positioned in the edentulous space of tooth 36. In addition, a peri-implantitis-like bone defect was created around the implant at site 36. When designing the defect-like bone morphology, the most frequently observed defect configurations were selected as described by Schwarz et al. [14]. Two different defect models were planned: a buccal dehiscence defect (Type 1) and a circumferential crater/fossa defect (Type 2). To represent peri-implantitis-like bone loss, a defect with a depth of 4 mm from the crestal level was prepared. All models were designed under identical standards in the virtual environment and subsequently manufactured using a 3D printer (Figure 1).

A Phrozen Sonic Mini 8K (MSLA/LCD) 3D printer (Phrozen Tech Co., Ltd., Hsinchu City, Taiwan [R.O.C.]) was used to fabricate the models. Three-dimensional printing was performed using ARMA Model Resin, a photopolymer resin suitable for model fabrication (Arma Dental, Istanbul, Turkey). The printed models were washed with isopropyl alcohol in accordance with the manufacturer’s recommendations and polymerized for 10 min in the same company’s curing device, ensuring uniform light exposure from all directions. The models were stored for 1 day in a closed room with adequate ventilation, after which the stained implants were placed into the models.

### 2.4. Staining of Implants

**Figure 2:**
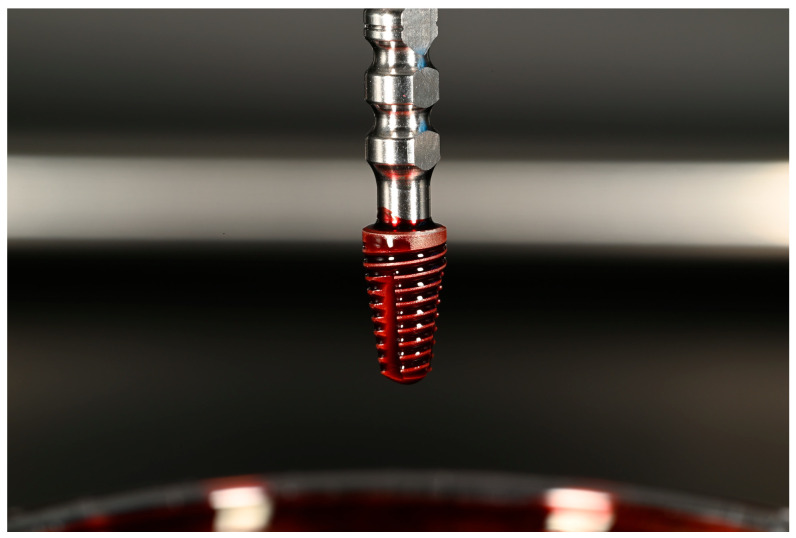
Implant surface painted with completely permanent ink.

Bone-level Zinedent implants (Zinedent, Instradent AG, Peter Merian-Weg 12, CH-4052, Basel, Switzerland) manufactured from grade 4 titanium with an SLA (sandblasted, large-grit, acid-etched) surface were used (3.5 mm diameter, 10 mm length). No machined or polished collar surface was present at the implant neck. To simulate a plaque-covered surface, 32 implants were immersed for 5 s in red permanent ink (Staedtler permanent Lumocolor, Nuremberg, Germany), ensuring complete staining of the entire implant surface. The implants were then left to dry at room temperature for 24 h in a well-ventilated room, without additional drying agents, ensuring no exposure to further contamination. All regions of the implant surface were fully and homogeneously covered (Figure 2).

### 2.5. Air-Abrasive Procedures

Among the air-abrasion systems commonly used in clinical practice, the portable AIRFLOW^®^ Handy 3.0 device (EMS Electro Medical Systems S.A., Nyon, Switzerland), which can be attached to the dental unit, was selected [15,16,17].

AIRFLOW^®^ Classic Comfort Powder (EMS Electro Medical Systems S.A., Nyon, Switzerland), a sodium bicarbonate-based powder compatible with the air-abrasive system from the same manufacturer, was selected. The mean particle size of the sodium bicarbonate powder is approximately 40 µm. Sodium bicarbonate has long been used in dentistry and is one of the most extensively investigated air-abrasive powders in the literature [15,16,17,18,19].

In the manufacturer’s clinical use recommendations for AIRFLOW, technical points are explicitly stated, including a 30–60° angulation, a 3–5 mm working distance, continuous movement, directing the jet toward suction, and avoiding a perpendicular orientation to the surface. Accordingly, an experienced operator in air-abrasion procedures (Özay F.) performed the water–powder spray procedure for a total of 120 s, holding the nozzle at an average angle of 45°, at a distance of 3–4 mm, while rotating the models continuously by hand. Decontamination was applied to each accessible surface area as permitted by the model. After treatment, each model was gently dried with air spray without pressure to prevent cross-contamination, placed into clean autoclave pouches, and sealed tightly using an autoclave pouch sealer prior to storage.

### 2.6. Electrolytic Cleaning

**Figure 3:**
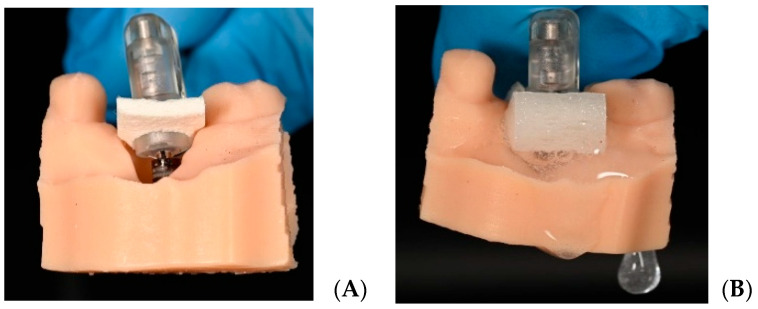
(**A**) Placement of the GalvoSurge device head into the implant. (**B**) Decontamination of the implant surface while the device is running. Small air bubbles are observed.

Implant surface decontamination was performed using the GalvoSurge^®^ Dental Implant Cleaning System control unit (GS 1000, GalvoSurge Dental AG, Widnau, Switzerland). The system consists of (I) a control unit, (II) a sterile single-use cleaning solution bottle, (III) a single-use tube package with a spray head at its distal end, and (IV) integrated components including an implant connector and a sponge. The cleaning solution bottle has a volume of 500 mL and is designed for single-patient/single-session use; according to the manufacturer, it provides sufficient volume for up to two implants in the same patient. In accordance with the manufacturer’s recommendations, each cleaning solution bottle was used for two models (Figure 3).

Technically, the device operates according to the “safety extra-low voltage” principle; the control unit provides a 15 V DC output voltage and a maximum output current of 0.6 A (600 mA). The pump flow rate is reported as 100 mL/min (±10%).

In the protocol described in the clinical literature for the electrolytic approach, the implant is charged as the cathode and the system delivers a sodium formate-based electrolyte solution via the spray head, separating the biofilm from the surface through hydrogen bubble formation [8].

After opening the sterile single-use Cleaning Solution and Tube Package, the components were connected to the control unit while avoiding contamination: the tubing was placed correctly into the pump housing, the solution bottle was connected via the spike, and the bottle was hung on the holding bar. The single-use components were not reused outside the same patient and the same session.

The spray head was seated onto the internal connection of the implant, and continuous electrical contact between the integrated implant connector and the implant was ensured by applying constant finger pressure on the head. Because loss of contact could interrupt cleaning, the stability of the head was maintained throughout the procedure. The system was initiated via the control unit. Initially, tube filling was awaited to allow the solution to pass from the bottle to the spray head; thereafter, the cleaning phase was started with electrolytic activation. The operator (F.Ö.) confirmed that the sponge became saturated with solution, expanded, and extended into deeper regions of the implant. Continuous solution flow and the formation of gas bubbles/foaming around the implant were observed; if solution flow was interrupted or bubble formation was not observed, the procedure was paused and the connections were checked.

For electrical safety and current distribution, metallic instruments were kept at least 20 mm away from the operative site. The electrolytic cleaning duration was applied as 2 min (120 s) in accordance with the manufacturer/device flow, and the device automatically terminated the procedure at the end of the cycle. After completion, the tube package and solution bottle were detached from the device and disposed of according to medical waste procedures. Residual solution around the implant was removed by aspiration.

### 2.7. Assessment of Implant Surfaces

For surface assessment, the implants were carefully removed from their respective models and transferred to the subsequent evaluation steps. Implants were first photographed and then analyzed using ImageJ visual analysis software (ImageJ 1.54) (National Institutes of Health, Bethesda, MD, USA).

#### 2.7.1. Photography of Implants

Each treated implant was gently removed from the artificial jaw model without contacting any other surface. The moist implant surface was dried using gentle low-pressure air. Implants were placed upside down into the original manufacturer’s carrier and positioned on a flat platform. Digital color photographs were obtained perpendicular to the implant axis using the standardized parameters described by Sharman et al. [20]. The standardized settings were ISO 100, aperture f/32, shutter speed 1/250 s, and a working distance of 31.4 cm [20]. Imaging was performed using a mirrorless Nikon Z5 camera body (Nikon, Tokyo, Japan) and a compatible 105 mm macro lens (NIKKOR Z MC 105 mm f/2.8 VR S). Photographs were acquired from one side of the implant and from the opposite side (after a 180° rotation).

To provide controlled and homogeneous illumination suitable for macro photography during photographic documentation of implant surfaces, the Godox MF12 Macro Twin Flash System (Godox, Shenzhen, China) was used. The MF12 macro flash unit was integrated with the mirrorless camera system positioned perpendicular to the implant axis, enabling visualization of surface details with high contrast and minimal shadowing.

#### 2.7.2. Measurement of Residual Stain on Implant Surfaces

**Figure 4:**
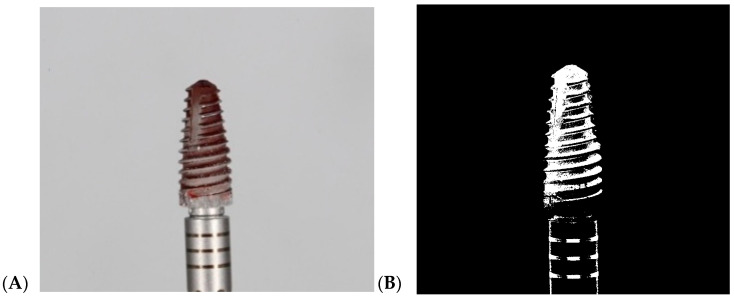

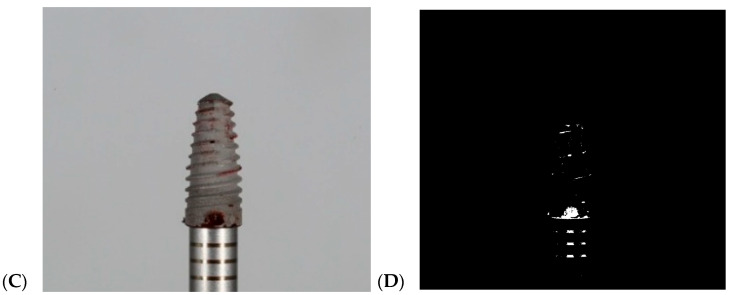
(**A**) Implant surface after air abrasion. (**B**) ImageJ image of the implant after air abrasion. (**C**) Implant surface after electrical decontamination. (**D**) ImageJ image of the implant after electrical decontamination.

Photographs of the implants after completion of the decontamination procedures were imported into ImageJ. For standardization, calibration was performed for each implant prior to measurement. Each implant used in the study (10 mm length and 3.5 mm diameter) was defined in the software, and calibration was achieved by matching real millimeters to pixels in the photographs. Because the analysis focused on the treated region, a 4 mm area extending apically from the neck region toward the implant body was selected. Under these settings, the stained area within the 4 mm region of a fully stained implant was measured and determined to be 0.2 mm^2^. Since all implants had the same diameter, length, and baseline stain coverage, the initial stain area was accepted as 0.2 mm^2^ for all implants. Residual stain percentage was calculated using the formula “% residue = (residual area × 100/total area)”, where the total area was taken as 0.2. For the remaining implants, the implant body was manually outlined using polygon selection and saved to maintain standardization. The residual stain area of each treated implant was then measured. For each implant, both surfaces were measured and recorded separately in the table; subsequently, the arithmetic mean of the two surfaces was calculated to obtain the final residual stain amount (Figure 4).

The ImageJ parameters and application procedure used in this study are consistent with similar analysis methods reported in the literature [19].

#### 2.7.3. SEM and EDS Imaging

After photography, the samples were placed into sterilization packages to prevent secondary contamination. The implants were sent on the same day to the TÜBİTAK Marmara Research Center and the SEM (Scanning Electron Microscope)/EDS (Energy Dispersive X-ray Spectroscopy) examination processes were initiated.

**Figure 5:**
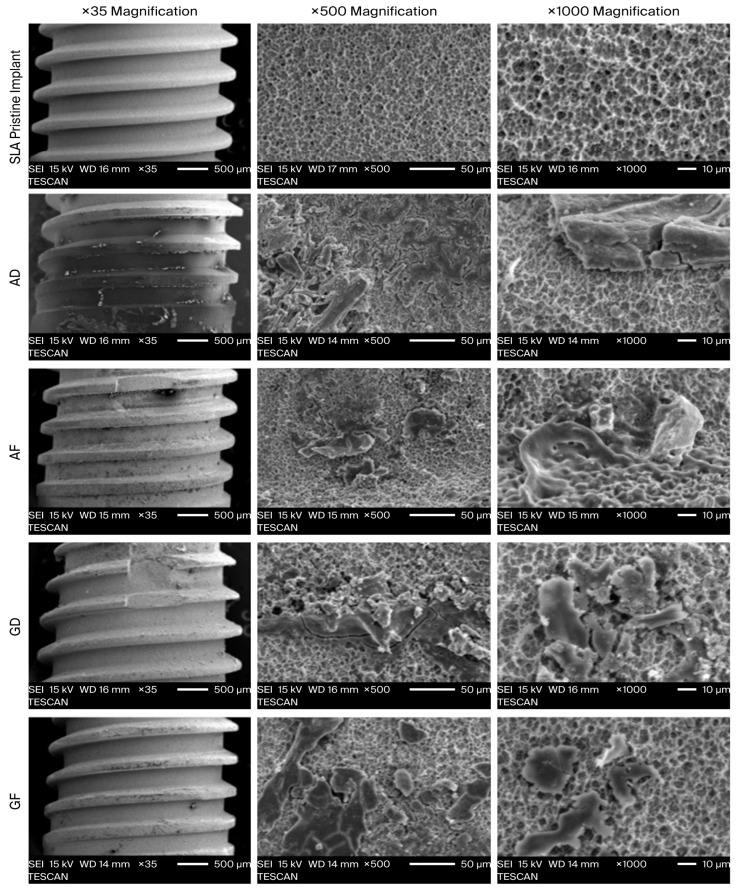
Scanning electron microscope images of the experimental surfaces. SLA surface revealed the typically known topographical features. **AD** (Airflow–Dehiscence defect), **AF** (Airflow–Fossa defect), **GD** (Galvosurge–Dehiscence defect), **GF** (Galvosurge–Fossa defect). NaHCO_3_ particles were observed as residue on the AD and AF surfaces. Permanent ink residues are also commonly present on the surface. In the GD and GF groups, lower remnant amounts were observed. The amount of residual dye was relatively less compared to the other groups.

Surface morphology of titanium implant specimens after decontamination and the evaluation of residual deposits were performed using the electron microscopy infrastructure of the TÜBİTAK Marmara Research Center (MAM) Materials Institute. Analyses were carried out using a JEOL, Tokyo, Japan, JSM-6335F field-emission scanning electron microscope (FE-SEM) integrated with an Oxford INCA energy-dispersive X-ray spectroscopy (EDS) system. Images were randomly taken from a 4 mm area below the neck region of the implants. Each row in the graph indicates the treatment performed and the type of defect. The first row shows images of untreated pure titanium. The first column shows ×35 magnification, the second column shows ×500 magnification, and the third column shows ×1000 magnification (Figure 5).

EDS analyses were conducted using the Oxford INCA software (**INCAMicroAnalysis version.**)/hardware platform; INCA-based EDS systems, typically enable elemental analysis with an energy resolution on the order of ~140 eV [21].

Surface topography was assessed using secondary electron imaging mode (SEI). Images were acquired at low magnification (×35) to visualize overall morphology and the distribution of deposits on implant threads, and at higher magnifications (×500 and ×1000) to examine micro-topographic details and deposit characteristics. Images were recorded from different regions at randomly selected, standardized points.

Following SEM imaging, EDS point analysis and elemental mapping were performed on the same specimens to chemically characterize the deposits. EDS measurements were planned to compare areas with dense deposits with reference areas of the implant that appeared relatively clean. During measurement, the combined use of EDS with SEM is considered an appropriate approach for distinguishing residual deposits (organic/inorganic) after decontamination, as it enables correlation of morphology and elemental composition within the same micro-region [22,23].

### 2.8. Statistical Analysis

Statistical analyses were performed using IBM SPSS v23 and Jamovi v2.7.6 software. First, the normality assumption was assessed for each variable prior to analysis using the Shapiro–Wilk test. Appropriate analytical methods were then selected depending on whether the data were normally distributed according to the model–treatment interaction. Robust ANOVA (robust analysis of variance), a non-parametric approach, was used for comparisons when the normality assumption was not met, whereas two-way ANOVA was applied when data conformed to normality. For descriptive statistics, quantitative data were summarized according to distribution characteristics as follows: mean ± standard deviation (for approximately normally distributed data), trimmed mean ± standard error (to reduce the influence of outliers), and median (minimum–maximum) (for non-symmetric distributions).

In all analyses, the level of statistical significance was set at *p* < 0.05, and *p*-values below this threshold were considered statistically significant.

## 3. Results

### 3.1. Findings of Photographic Analysis

All experiments were successfully completed. Samples were processed as planned, required examinations were performed, and results were recorded. Accordingly, all models were grouped as shown below, and the findings presented are described according to this grouping (Table 1).

Regardless of treatment, mean residual stain amount values did not differ statistically between models (*p* = 0.258). The mean value was 0.07 in Type 1 and 0.062 in Type 2. Regardless of model, mean residual stain amount values differed statistically between treatments (*p* = 0.019). The mean value was 0.074 in Treatment 1 and 0.057 in Treatment 2. According to the Model × Treatment interaction, mean residual stain amount values did not differ statistically (*p* = 0.365) (Table 2).

Regardless of treatment, mean residual stain percentage values did not differ statistically between models (*p* = 0.258). The mean value was 34.813 in Type 1 and 30.797 in Type 2. Regardless of model, mean residual stain percentage values differed statistically between treatments (*p* = 0.019). The mean value was 37.141 in Treatment 1 and 28.469 in Treatment 2. According to the Model × Treatment interaction, mean residual stain percentage values did not differ statistically (*p* = 0.365).

In the comparison based on the residual stain percentage variable calculated by ImageJ analysis, a clear separation in distribution between groups was observed. The Type 1–Treatment 1 group exhibited the highest residual stain percentage values (mean 40.75 ± 11.42; median 40.13), whereas lower mean values were found in the GalvoSurge-treated groups (GD: 28.88 ± 8.78; GF: 28.06 ± 11.94). In the two-factor analysis of variance, the method effect was significant (AIRFLOW vs. GalvoSurge, *p* = 0.0189; η^2^ = 0.171), whereas defect type and the method × defect interaction were not significant (*p* > 0.05). These findings indicate that the GalvoSurge method generally reduced the residual stain percentage, although residual contamination may vary across specimens (Table 3).

### 3.2. SEM Findings

In the untreated clean reference specimen, a homogeneous, sponge-like micro-porous topography was observed across the surface. An interconnected network of micro-pits and crater-like depressions was present, with regular inter-pore connections. No foreign particles or layering were observed; the voids were open and clearly distinguishable.

In the AIRFLOW-treated specimens, the presence of cracked, mud-like fractured plaques was notable. This suggests a shrinking organic–inorganic layer formed upon drying, indicative of surface contamination structures such as biofilm remnants or a dye-precipitated salt matrix. This layer obscured the underlying micro-porous titanium topography. Numerous elongated prismatic rod-like fragments and occasional flower-like clusters were observed. The contaminated structures observed on the surface are morphologically consistent with cleaning-agent residues, crystals precipitated from the medium, or debris that was loosened, displaced, and subsequently redeposited.

In the SEM images (×500) of the Treatment 2 group obtained after electrochemical cleaning with the GalvoSurge device and the original solution, the homogeneous micro-porous topography observed on a clean implant surface was not fully re-exposed in many specimens. Instead, the surface was covered to varying extents by residual layers and/or granular–crystalline particulate deposits. In exposed regions, the underlying roughness architecture was generally preserved, and prominent mechanical abrasion scratches were not dominant.

When groups were evaluated collectively, the overall morphological comparison at SEM magnifications (×500 and ×1000) indicated that in AIRFLOW-treated specimens (AD [treatment1–type1]/AF [treatment1–type2]) residual contamination was predominantly observed as granular particle clusters and island-like film fragments, and the micro-rough titanium topography became discernible over broader areas, particularly in the AF subgroup.

In contrast, following GalvoSurge (GD [treatment2–type1]/GF [treatment2–type2]), residual structures more frequently appeared as thicker film-like layers, sometimes accompanied by crust-like deposits, covering the surface more extensively and increasingly masking micro-topography. Overall, in the galvanic current decontamination (Treatment 2) group, the pattern was more variable and more consistent with film-character residual contamination than with AIRFLOW.

### 3.3. EDS Findings

Regardless of treatment, median Na values did not differ statistically between models (*p* = 0.648). The median value was 2.14 in Type 1 and 2.92 in Type 2. Regardless of model, median Na values did not differ statistically between treatments (*p* = 0.325). The median value was 3.773 in Treatment 1 and 1.34 in Treatment 2. According to the Model × Treatment interaction, median Na values did not differ statistically (*p* = 0.643) (Table 4).

Regardless of treatment, median Ca values did not differ statistically between models (*p* = 0.977). The median value was 0 in both Type 1 and Type 2. Regardless of model, median Ca values did not differ statistically between treatments (*p* = 0.854). The median value was 0 in Treatment 1 and 0.22 in Treatment 2. According to the Model × Treatment interaction, median Ca values did not differ statistically (*p* = 0.942) (Table 5).

Regardless of treatment, median Ti values did not differ statistically between models (*p* = 0.49). The median value was 9.033 in Type 1 and 10.385 in Type 2. Regardless of model, median Ti values did not differ statistically between treatments (*p* = 0.084). The median value was 6.353 in Treatment 1 and 23.61 in Treatment 2. According to the Model × Treatment interaction, median Ti values did not differ statistically (*p* = 0.299) (Table 6).

Regardless of treatment, median Si values did not differ statistically between models (*p* = 0.767). The median value was 0 in both Type 1 and Type 2. Regardless of model, median Si values did not differ statistically between treatments (*p* = 0.074). The median value was 0.405 in Treatment 1 and 0 in Treatment 2. According to the Model × Treatment interaction, median Si values did not differ statistically (*p* = 0.668) (Table 7).

Regardless of treatment, mean Al values did not differ statistically between models (*p* = 0.547). The mean value was 0.015 in Type 1 and 0.043 in Type 2. Regardless of model, mean Al values did not differ statistically between treatments (*p* = 0.236). The mean value was 0.058 in Treatment 1 and 0 in Treatment 2. According to the Model × Treatment interaction, mean Al values did not differ statistically (*p* = 0.547) (Table 8).

Regardless of treatment, mean Cl values did not differ statistically between models (*p* = 0.145). The mean value was 0.259 in Type 1 and 1.089 in Type 2. Regardless of model, mean Cl values differed statistically between treatments (*p* = 0.045). The mean value was 0.076 in Treatment 1 and 1.272 in Treatment 2. According to the Model × Treatment interaction, mean Cl values did not differ statistically (*p* = 0.09) (Table 9).

Regardless of treatment, mean K values did not differ statistically between models (*p* = 0.104). The mean value was 0.015 in Type 1 and 0.165 in Type 2. Regardless of model, mean K values did not differ statistically between treatments (*p* = 0.104). The mean value was 0.015 in Treatment 1 and 0.165 in Treatment 2. According to the Model × Treatment interaction, mean K values did not differ statistically (*p* = 0.057) (Table 10).

Regardless of treatment, mean S values did not differ statistically between models (*p* = 0.348). The mean value was 0.929 in Type 1 and 0 in Type 2. Regardless of model, mean S values did not differ statistically between treatments (*p* = 0.348). The mean value was 0 in Treatment 1 and 0.929 in Treatment 2. According to the Model × Treatment interaction, mean S values did not differ statistically (*p* = 0.348) (Table 11).

Regardless of treatment, mean Cr values did not differ statistically between models (*p* = 0.661). The mean value was 0.158 in Type 1 and 0.078 in Type 2. Regardless of model, mean Cr values did not differ statistically between treatments (*p* = 0.207). The mean value was 0 in Treatment 1 and 0.236 in Treatment 2. According to the Model × Treatment interaction, mean Cr values did not differ statistically (*p* = 0.661) (Table 12).

Regardless of treatment, mean Fe values did not differ statistically between models (*p* = 0.348). The mean value was 0 in Type 1 and 0.078 in Type 2. Regardless of model, mean Fe values did not differ statistically between treatments (*p* = 0.348). The mean value was 0 in Treatment 1 and 0.078 in Treatment 2. According to the Model × Treatment interaction, mean Fe values did not differ statistically (*p* = 0.348) (Table 13).

## 4. Discussion

**Figure 6:**
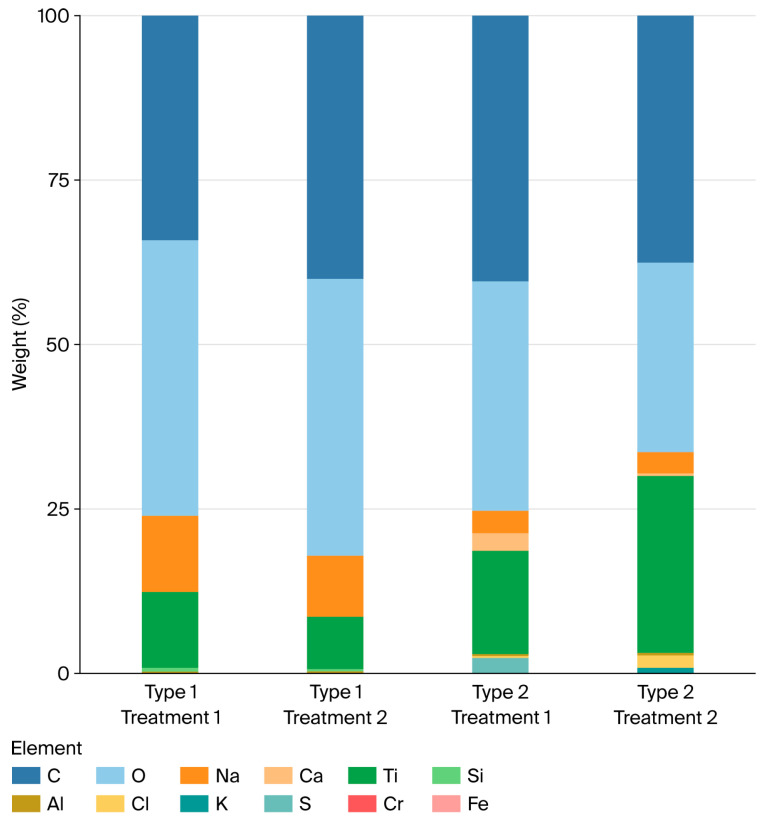
Percentages of elements remaining (weight %) on implant surfaces as a result of EDS imaging.

In this study, two different decontamination approaches (AIRFLOW + sodium bicarbonate; GalvoSurge + original electrolyte solution) were compared on micro-rough titanium implant surfaces. After the two treatments, the amount of residual stain (%) and surface topography were evaluated using SEM (×500–×1000) and EDS (Figure 6). In the quantitative analysis, the method effect was significant; the residual stain percentage was lower in the GalvoSurge-treated groups than in the AIRFLOW groups (AIRFLOW vs. GalvoSurge *p* = 0.0189; η^2^ = 0.171).

In our study, specimens treated with AIRFLOW + sodium bicarbonate generally displayed a heterogeneous cleaning pattern: while the micro-porous titanium texture became discernible again in some areas, cracked film layers masking pores and particle deposits with variable morphologies were observed in most regions.

These morphological findings are consistent with the potential of sodium bicarbonate to provide effective mechanical debridement while still leaving residual particles/films. Indeed, recent in vitro studies have shown that air-polishing with sodium bicarbonate can be effective in reducing biofilm; however, it may also generate particle deposits/elemental accumulations and changes in surface roughness [24,25].

The clinical literature on GalvoSurge has been shaped by randomized controlled trials reporting that electrolytic cleaning, when combined with a surgical regenerative approach, can yield improvements in clinical parameters and outcomes such as radiographic bone fill [11].

In our study, mean residual stain percentage values based on ImageJ analysis were higher in AD and AF and lower in GD and GF (AD: 40.75 ± 11.42; AF: 33.53 ± 6.11; GD: 28.88 ± 8.78; GF: 28.06 ± 11.94). This quantitative result suggests that GalvoSurge may be advantageous for stain removal. However, the observation of more compact film-layer residues after GalvoSurge on SEM indicates that a reduction in stain does not always parallel an increase in micro-topographic openness.

A single-center randomized controlled pilot study by Alberto Monje et al. compared electrolytic cleaning (GalvoSurge) with 3% hydrogen peroxide for implant surface decontamination during reconstructive surgery in peri-implantitis defects. At 12 months, both approaches yielded clinical and radiographic improvements and showed similar marginal bone gain, while disease resolution was higher in the electrical cleaning group; however, due to sample size limitations, statistical superiority could not be definitively established. The findings suggest that treatment outcomes depend not only on the decontamination technique but also on prognostic factors such as baseline probing depth and intrabony component depth, as well as the overall regenerative protocol. While supporting electrical cleaning as a safe and effective alternative, the study highlights the need for confirmation in stronger trials [26].

In the 2023 study by Assunção et al., 20 SLA implants were contaminated with *P. aeruginosa* biofilm and compared across an electrolytic method, an erythritol jet system (PerioFlow), and two titanium brushes (R-Brush/i-Brush). Except for R-Brush, the methods showed similar biofilm removal performance, whereas major surface alterations on SEM were reported particularly for titanium brushes. This pilot study, similar to ours, is valuable for discussing the balance between efficacy and surface damage: it indicates that air-abrasive/erythritol and electrolytic approaches may better preserve surface topography, whereas metal brushes may induce surface modification, providing a literature basis when interpreting our SEM/EDS findings in terms of whether the cost of cleaning translates into surface damage [8].

In a study by Ratka et al., electrolytic cleaning was compared with the “air-powder-water spray (PSS)” approach; sterile implants were placed in an incubator to form a biofilm. After electrolytic cleaning and powder spray system cleaning, the implants were transferred to Eppendorf tubes containing culture medium. As a result, a total of 210 blood agar plaques were evaluated in the electrolytic cleaning groups, and not a single CFU (Colony Forming Unit) was detected. However, intense growth was reported in the control method. This study answers the question of whether the electrolytic method is superior to the mechanical/air spray method by using a direct microbiological outcome point. However, while that study measured cleaning in CFU (Colony Forming Unit), our study is limited to a morphological and quantitative approach to the removal of visible stained residues [27].

The contribution of our study to the literature is that beyond clinical endpoints, it renders residual morphology visible through a detailed SEM comparison and links this, together with defect geometry, to a quantitative cleaning metric (residual stain %). Notably, the significance of the method effect in the absence of a significant defect type × method interaction reinforces the idea that, in clinical scenarios with different defect morphologies, absolute superiority of a single method may be less decisive than procedural standardization and residue management.

The multi-modal assessment approach in this study (ImageJ + SEM + EDS) enabled evaluation of cleaning efficacy without reducing it to a single metric, integrating quantitative (residual stain) and qualitative (residue morphology) differentiation. The design incorporating defect geometry—comparing dehiscence and fossa models—allowed experimental modeling of access/flow-dynamics differences frequently encountered clinically.

Despite these strengths, one aspect requiring improvement is the inability of the in vitro model to replicate biological complexity. Our study focused on the removal of dye residue contamination around implants; no biological validation could be performed, and no results were obtained in this regard. In clinical peri-implantitis lesions, variables such as blood, salivary proteins, true biofilm matrix, and mechanical access are more complex [28]; therefore, in vitro ink contamination may not fully represent biofilm behavior. This limitation is also frequently emphasized in the literature when discussing the clinical generalizability of in vitro air-polishing studies [29].

In our study, permanent ink was used to simulate contamination around the implant. While removing this permanent ink from the implant surface gives us an idea of the cleaning capacity of the cleaning methods we compared, it is primarily aimed at evaluating dye and film removal. Periimplantitis implant surfaces, exposed to many bacterial species and saliva in the oral environment, contain various structures such as multispecies biofilm, calculus, and endotoxin. Removing these structures from the implant surface is much more complex and difficult than permanent dye removal procedures. Therefore, the promising results of the permanent dye removal and residue removal process in our study may not be equivalent to biofilm removal and definitive cure of periimplantitis in clinical practice. Our study on the removal of visible contamination and stained residues should be supported by clinical studies, building upon the limited data from in vitro studies.

In addition to these limitations, there is a need for a head-to-head morphological comparison of AIRFLOW and GalvoSurge under the same framework and at the same SEM magnifications. The number of studies comparing these two approaches directly using a standardized SEM typology is limited; thus, our study contributes directly to this gap and provides a basis for discussion regarding where each method may be more predictable.

For future studies, it is recommended and necessary to use standardized in vitro multi-species biofilms instead of ink in the same model and to support outcomes with microbiological metrics (CFU/qPCR). In addition, verifying EDS findings with more sensitive methods for surface chemistry, such as profilometry (Sa/Ra) and XPS, is advised.

## 5. Conclusions

GalvoSurge is an effective and clinically feasible decontamination method. When assessed based on the percentage of residual stain on implant surfaces after decontamination, it leaves a lower residue compared with AIRFLOW. On titanium implant surfaces, galvanic current-based cleaning produces less damage than air-abrasion systems. For AIRFLOW, the composition and particle size of the powder are important with respect to cleaning capacity and the amount of residue left on implant surfaces. The difficulty of applying air-abrasive devices in a standardized manner represents a disadvantage relative to GalvoSurge. With respect to peri-implantitis defect configurations, no significant difference was observed in terms of implant decontamination.

This study makes the morphology of residual deposits visible through SEM-based comparison and relates these findings to a quantitative cleaning metric, namely, the percentage of remaining stain, together with the factor of defect geometry (dehiscence/fossa). In particular, the finding that the treatment method had a statistically significant effect whereas the defect type × method interaction was not significant strengthens the notion that, in clinical practice, rather than indicating the absolute superiority of a single method across different peri-implant bone defect morphologies, the standardization of the application protocol and the management of residual deposits may be more decisive determinants of decontamination outcomes.

In the present study, by employing a multimodal assessment strategy, cleaning efficacy was not reduced to a single parameter; instead, both quantitative findings (percentage of remaining stain) and qualitative findings (residual morphology) were evaluated in an integrated manner. Nevertheless, despite these strengths, the fact that contamination was established solely by means of permanent dye limits the direct clinical applicability of the results. For future investigations, repetition of the same experimental model using standardized in vitro multispecies biofilm instead of dye, and support of the findings with microbiological metrics such as CFU and qPCR, are recommended. Furthermore, confirmation of the EDS findings by more sensitive surface characterization techniques, such as profilometry (Sa/Ra) and XPS, would further strengthen the scientific value of the study. Overall, these data indicate the need for more comprehensive future studies.

## Figures and Tables

**Table 1:** Implant groups.

Defects/Decontaminations	Air Abrazive Decontamination Airflow (A) (Treatment 1)	Eleyctrical Decontamination GALVOSurge (G) (Treatment 2)
(D) Dehiscence (Type 1)	8 implants	8 implants
(F) Fossa (Type 2)	8 implants	8 implants

**Table 2:** Comparison of residual stain amount values according to models and treatments.

Treatment	Model		Total		Test Statistic	*p*	KEK
	Type 1	Type 2					
Treatment 1	0.082 ± 0.023	0.067 ± 0.012	0.074 ± 0.019	Model	1.332	0.258	0.045
Treatment 2	0.058 ± 0.018	0.056 ± 0.024	0.057 ± 0.02	Treatment	6.214	0.019	0.182
Total	0.07 ± 0.023	0.062 ± 0.019		Model × Treatment	0.848	0.365	0.029

Two-way ANOVA; Mean ± Standard Deviation; PES: Partial Eta Squared (η^2^).

**Table 3:** Comparison of residual stain percentage values according to models and treatments.

Treatment	Model		Total		Test Statistic	*p* ^x^	KEK
	Tip 1	Tip 2					
Treatment 1	40.75 ± 11.418	33.531 ± 6.113	37.141 ± 9.601	Model	1.332	0.258	0.045
Treatment 2	28.875 ± 8.778	28.063 ± 11.937	28.469 ± 10.131	Treatment	6.214	0.019	0.182
Total	34.813 ± 11.593	30.797 ± 9.587		Model × Treatment	0.848	0.365	0.029

Two-way ANOVA; Mean ± Standard Deviation; PES: Partial Eta Squared (η^2^).

**Table 4:** Comparison of Na (Weight) values according to models and treatments.

Treatment	Model		Total		Test Statistic	*p* ^x^	KEK
	Type 1	Type 2					
Treatment 1	7.858 (0.62–32.195)	3.773 (0.325–29.61)	3.773 (0.325–32.195)	Model	0.208	0.648	0.007
Treatment 2	1.385 (0.16–15.16)	1.34 (0–9.58)	1.34 (0–15.16)	Treatment	0.969	0.325	0.033
Total	2.14 (0.16–32.195)	2.92 (0–29.61)		Model × Treatment	0.214	0.643	0.008

Robust ANOVA (median used as the comparison metric); Median (Minimum–Maximum); PES: Partial Eta Squared (η^2^).

**Table 5:** Comparison of Ca (Weight) values according to models and treatments.

Treatment	Model		Total		Test Statistic	*p* ^x^	KEK
	Type 1	Type 2					
Treatment 1	0 (0–1.56)	0 (0–0.66)	0 (0–1.56)	Model	0.000	0.977	0.000
Treatment 2	0.3 (0–14.37)	0.22 (0–1.87)	0.22 (0–14.37)	Treatment	0.034	0.854	0.001
Total	0 (0–14.37)	0 (0–1.87)		Model × Treatment	0.005	0.942	0.000

Robust ANOVA (median used as the comparison metric); Median (Minimum–Maximum); PES: Partial Eta Squared (η^2^).

**Table 6:** Comparison of Ti (Weight) values according to models and treatments.

Treatment	Model		Total		Test Statistic	*p* ^x^	KEK
	Type 1	Type 2					
Treatment 1	5.965 (2.25–31.475)	7.315 (3.2–14.63)	6.353 (2.25–31.475)	Model	0.477	0.490	0.017
Treatment 2	15.48 (0–33.31)	25 (2.34–69.29)	23.61 (0–69.29)	Treatment	2.986	0.084	0.096
Total	9.033 (0–33.31)	10.385 (2.34–69.29)		Model × Treatment	1.080	0.299	0.037

Robust ANOVA (median used as the comparison metric); Median (Minimum–Maximum); PES: Partial Eta Squared (η^2^).

**Table 7:** Comparison of Si (Weight) values according to models and treatments.

Treatment	Model		Total		Test Statistic	*p* ^x^	KEK
	Type 1	Type 2					
Treatment 1	0.405 (0–1.135)	0.29 (0–1.03)	0.405 (0–1.135)	Model	0.088	0.767	0.003
Treatment 2	0 (0–1.09)	0 (0–0.69)	0 (0–1.09)	Treatment	3.194	0.074	0.102
Total	0 (0–1.135)	0 (0–1.03)		Model × Treatment	0.184	0.668	0.007

Robust ANOVA (median used as the comparison metric); Median (Minimum–Maximum); PES: Partial Eta Squared (η^2^).

**Table 8:** Comparison of Al (Weight) values according to models and treatments.

Treatment	Model		Total		Test Statistic	*p* ^x^	EB
	Type 1	Type 2					
Treatment 1	0.029 ± 0.033	0.087 ± 0.097	0.058 ± 0.05	Model	0.393	0.547	0.044
Treatment 2	0 ± 0	0 ± 0	0 ± 0	Treatment	1.607	0.236	0.074
Total	0.015 ± 0.016	0.043 ± 0.048		Model × Treatment	0.393	0.547	0.044

Robust ANOVA (trimmed mean used as the comparison metric); Trimmed Mean ± Standard Error; ES: Robust Bootstrap Effect Size.

**Table 9:** Comparison of Cl (Weight) values according to models and treatments.

Treatment	Model		Total		Test Statistic	*p* ^x^	EB
	Type 1	Type 2					
Treatment 1	0.152 ± 0.111	0 ± 0	0.076 ± 0.058	Model	2.550	0.145	0.114
Treatment 2	0.366 ± 0.266	2.177 ± 1.117	1.272 ± 0.612	Treatment	5.310	0.045	0.211
Total	0.259 ± 0.143	1.089 ± 0.623		Model × Treatment	3.580	0.090	0.151

Robust ANOVA (trimmed mean used as the comparison metric); Trimmed Mean ± Standard Error; ES: Robust Bootstrap Effect Size.

**Table 10:** Comparison of K (Weight) values according to models and treatments.

Treatment	Model		Total		Test Statistic	*p* ^x^	EB
	Type 1	Type 2					
Treatment 1	0.03 ± 0.033	0 ± 0	0.015 ± 0.017	Model	3.300	0.104	0.128
Treatment 2	0 ± 0	0.33 ± 0.18	0.165 ± 0.099	Treatment	3.300	0.104	0.128
Total	0.015 ± 0.017	0.165 ± 0.099		Model × Treatment	4.760	0.057	0.167

Robust ANOVA (trimmed mean used as the comparison metric); Trimmed Mean ± Standard Error; ES: Robust Bootstrap Effect Size.

**Table 11:** Comparison of S (Weight) values according to models and treatments.

Treatment	Model		Total		Test Statistic	*p* ^x^	EB
	Type 1	Type 2					
Treatment 1	0 ± 0	0 ± 0	0 ± 0	Model	1.000	0.348	0.058
Treatment 2	1.86 ± 2.07	0 ± 0	0.929 ± 1.03	Treatment	1.000	0.348	0.058
Total	0.929 ± 1.03	0 ± 0		Model × Treatment	1.000	0.348	0.058

Robust ANOVA (trimmed mean used as the comparison metric); Trimmed Mean ± Standard Error; ES: Robust Bootstrap Effect Size.

**Table 12:** Comparison of Cr (Weight) values according to models and treatments.

Treatment	Model		Total		Test Statistic	*p* ^x^	EB
	Type 1	Type 2					
Treatment 1	0 ± 0	0 ± 0	0 ± 0	Model	0.204	0.661	0.040
Treatment 2	0.315 ± 0.35	0.156 ± 0.174	0.236 ± 0.19	Treatment	1.796	0.207	0.078
Total	0.158 ± 0.175	0.078 ± 0.087		Model × Treatment	0.204	0.661	0.040

Robust ANOVA (trimmed mean used as the comparison metric); Trimmed Mean ± Standard Error; ES: Robust Bootstrap Effect Size.

**Table 13:** Comparison of Fe (Weight) values according to models and treatments.

Treatment	Model		Total		Test Statistic	*p* ^x^	EB
	Type 1	Type 2					
Treatment 1	0 ± 0	0 ± 0	0 ± 0	Model	1.000	0.348	0.057
Treatment 2	0 ± 0	0.155 ± 0.172	0.078 ± 0.086	Treatment	1.000	0.348	0.057
Total	0 ± 0	0.078 ± 0.086		Model × Treatment	1.000	0.348	0.057

Robust ANOVA (trimmed mean used as the comparison metric); Trimmed Mean ± Standard Error; ES: Robust Bootstrap Effect Size.

## Data Availability

The original contributions presented in this study are included in the article. Further inquiries can be directed to the corresponding authors.
